# Bacterial bloodstream infections in pediatric oncology patients in a tertiary medical center: pathogen distribution, antimicrobial nonsusceptibility, and outcomes

**DOI:** 10.1007/s00431-026-07305-7

**Published:** 2026-08-12

**Authors:** Menucha Jurkowicz, Eugene Leibovitz, Hila Rosental, Nathan Keller, Gilad Sherman, Efrat Dresner, Guy Katzenellenbogen, Elina Gelman, Michal Paret, Reut Myaskovetzky-Yitshack, Hana Golan, Assaf Barg, Amos Toren, Michal Stein

**Affiliations:** 1 Pediatric Infectious Disease Unit, The Edmond and Lily Safra Children’s Hospital, Sheba Medical Center, Ramat Gan, Israel; 2 Department of Epidemiology and Preventive Medicine, School of Public Health, Gray Faculty of Medicine, Tel Aviv University, Tel Aviv, Israel; 3 Faculty of Health Sciences, Ben-Gurion University, Beer Sheva, Israel; 4 Gray Faculty of Medical and Health Sciences, Tel Aviv University, Tel Aviv, Israel; 5 The Department of Health Management, Ariel University, Ariel, Israel; 6 Division of Infectious Diseases, Department of Pediatrics, Boston Children’s Hospital, Harvard Medical School, Boston, MA USA; 7 Clinical Pharmacy Unit, Sheba Medical Center, Ramat Gan, Israel; 8 Division of Pediatric Hematology, Oncology, and Bone Marrow Transplant, Edmond and Lily Safra Children’s Hospital, Sheba Medical Center, Ramat Gan, Israel

**Keywords:** Bloodstream infection, Pediatric, Hemato-oncology, Bacteremia, Neutropenia

## Abstract

The purpose of this study was to analyze bacterial bloodstream infections (BSIs) occurring in hospitalized pediatric hemato-oncology patients during 2012 to 2023. Data on 775 bacterial BSI episodes occurring in 456 patients (292, 63.04%, hematological malignancies and 164, 35.96%, solid tumors) were retrospectively collected and analyzed. In total, 362 (46.71%) bacterial-BSIs had profound neutropenia; 849 pathogens were isolated; gram-negative (GN) (68.78%, 584/849) were more common than gram-positive (GP) pathogens (30.86%, 262/849), *P* < 0.001. The most frequent GN isolates were *Klebsiella* spp. (20.38% of all isolates), *Escherichia coli* (19.20%), and *Pseudomonas aeruginosa* (12.01%); the most common GP isolates were *Streptococcus viridans* (9.19%) *Enterococcus* spp. (6.36%), and *Staphylococcus aureus* (6.12%, 21.15% MRSA). An increase in GN rates was observed in 2018–2023 versus 2012–2017 (74.16% vs. 63.22%, *P* = 0.001). Overall nonsusceptibility rates of *Klebsiella* spp. and *E. coli* were 80.13% and 90.07% for piperacillin, 16.76% and 9.82% for amikacin, 69.19% and 50.31% for ceftriaxone, 34.68% and 23.92% for piperacillin/tazobactam, and 8.72% and 1.85% for meropenem. Nonsusceptibility rates of *Acinetobacter baumannii* were 57.14% for both piperacillin/tazobactam and meropenem. The appropriateness of empiric antibiotic therapy (EAT) with piperacillin/tazobactam was lower than piperacillin/tazobactam + amikacin (62.61% vs. 90.45%, *P* < 0.0001). PICU admission was required in 230 (29.68%); 7-day and 30-day mortality rates were 6.36% and 14.69%, respectively.

*Conclusions*:

We report an increase in GN-BSIs and in nonsusceptibility rates of *Klebsiella* spp. and *E. coli* to several first/second-line antibiotics used in empiric antibiotic therapy. In our setting, an EAT protocol including piperacillin/tazobactam and amikacin covered most pathogens and should be considered in stable children with suspicion of BSI.

**Table:** 

**What is Known:** • *Bloodstream infections and antibiotic resistance rates are increasing in pediatric hemato-oncology patients.*	
**What is New:** • *We found a dominance of gram-negative pathogens and moderate to high resistance rates of Klebsiella spp. and Escherichia coli for piperacillin/tazobactam, ciprofloxacin, and ceftriaxone.* • *Empiric antibiotic therapy was appropriate for 77.07% of evaluable pathogens.* • *The appropriateness of empiric antibiotic therapy with piperacillin/tazobactam alone was lower than that of piperacillin/tazobactam plus amikacin.*

**What is Known:**

• *Bloodstream infections and antibiotic resistance rates are increasing in pediatric hemato-oncology patients.*

**What is New:**

• *We found a dominance of gram-negative pathogens and moderate to high resistance rates of Klebsiella spp. and Escherichia coli for piperacillin/tazobactam, ciprofloxacin, and ceftriaxone.*

• *Empiric antibiotic therapy was appropriate for 77.07% of evaluable pathogens.*

• *The appropriateness of empiric antibiotic therapy with piperacillin/tazobactam alone was lower than that of piperacillin/tazobactam plus amikacin.*

## Introduction

Bloodstream infections (BSIs) occur in up to 50% of pediatric cancer patients [1–6] and the characteristics of BSI acquisition and mortality can be cancer-specific [3–5, 7, 8]. In particular, BSIs are more frequent in patients with acute myeloid leukemia (AML) and during induction therapy, while BSI characteristics differ between hematologic malignancies and solid tumors [9–11]. The choice of empiric antibiotic treatment (EAT) for suspected BSI must consider the patient’s clinical status including previous colonization with relevant pathogens, predominant local pathogens, and regional antimicrobial susceptibility patterns [3, 4]. In recent decades, the epidemiology of BSIs in hemato-oncology patients has changed considerably, with a significant decrease in gram-positive (GP) pathogens and an increase in gram-negative (GN) bacteria [12–15]. More recently, a progressive rise in multidrug-resistant (MDR) strains has been reported [16–18]. In Europe, a 2014 survey from 39 hematology centers reported infection incidence rates of 15–24% for extended-spectrum β-lactamase-producing (ESBL) *Enterobacteriaceae*, 5–14% for aminoglycoside-resistant GN bacteria, and 5–14% for carbapenem-resistant *Pseudomonas aeruginosa* [19]. In a multicenter cohort study including 811 GN BSI episodes in Italian patients, Trecarichi et al. [20] reported a significant reduction in use of fluoroquinolone prophylaxis and a significant increase in susceptibility rates to ciprofloxacin.

Antibiotic resistance adversely affects survival among patients with hematological malignancies and hematopoietic stem cell transplant (HSCT) recipients [8, 15, 18–21]. O’Connor et al. reported a 30-day mortality rate of 3% among pediatric cancer patients with BSIs [22], while Willis et al. [23] reported a mortality rate of 6.5% in pediatric oncologic/HSCT patients with BISs compared with 0.7% in patients without BISs.

The high incidence of BSIs in hemato-oncology patients and the emergence of antibiotic resistance have led to an increased use of broad-spectrum antibiotics, including carbapenems, either as monotherapy or as combination therapy [3, 4, 8]. Knowledge of the local epidemiology of BSIs is pivotal in guiding the correct selection of antibiotic therapy in pediatric cancer patients. Local epidemiology should be incorporated into institutional guidelines and standard operating procedures that guide EAT at the time of initial presentation.

In this study, we aimed to evaluate the epidemiology, microbiology, antimicrobial susceptibility patterns, EAT management, and outcomes of bacterial BSIs in pediatric hemato-oncology patients during a 12-year period (2012–2023).

## Patients and methods

We retrospectively analyzed bacterial BSI episodes diagnosed between 2012 and 2023 in oncologic children aged < 18 years at Sheba Medical Center (a 2000-bed, tertiary, university-affiliated hospital in Israel). The study was approved by the Sheba Medical Center Institutional Review Board (SMC-D-0915–23).

The type of underlying disease was classified as follows: (1) hematologic malignancies including acute lymphoblastic leukemia (ALL), AML, Hodgkin’s disease, non-Hodgkin lymphoma, and other leukemias and (2) solid tumors including neuroblastoma, bone or soft tissue sarcoma, central nervous system tumor, and other solid tumors.

Inclusion criteria were a diagnosis of BSI (due to GP and GN pathogens or *Mycobacteria* spp.) in hemato-oncology patients treated with chemotherapy or allogeneic HSCT. In cases of suspected infection, two sets of blood samples had been drawn and processed in the microbiology laboratories. Regarding coagulase-negative staphylococci, *Micrococcus* species, and other skin contaminants, at least 2 consecutive sets of positive blood cultures were required to confirm a BSI [24].

### Clinical management

In each episode, demographic, clinical, microbiology, management, and outcome data were retrieved using MDClone ADAMS platform, a self-service query tool providing comprehensive patient-level data of wide-ranging variables in a defined timeframe around an index event (mdclone.com). The first-line dedicated EAT protocol for hemato-oncology patients admitted from the community with suspected BSI, including fever and neutropenia, was piperacillin plus amikacin or piperacillin/tazobactam from 2012 to 2015 and piperacillin/tazobactam plus amikacin for the period 2016–2023. The second-line EAT dedicated protocol was meropenem, prescribed in patients who developed signs of breakthrough infection during treatment with first-line protocol or in patients who appeared to be in severe sepsis.

The patients received antineoplastic therapy according to international protocols and recommendations, and the only antibiotic prophylaxis used was anti-*Pneumocystis jiroveci* prophylaxis with trimethoprim-sulfamethoxazole (twice daily, thrice weekly) which was routinely administered during the study period to all children with malignancies and after engraftment post-HSCT.

### Definitions

Fever was defined as a single-temperature measurement > 38.0°C at admission. Neutropenia was defined as ≤ 0.5 × 10^9^9/L, and profound neutropenia was defined as ≤ 0.1 × 10^9^/L.

BSI was defined as the detection of true pathogen bacteria in 1 or more blood cultures and polymicrobial BSIs were defined when ≥ 2 true pathogens were detected in a single blood culture.

For pathogen susceptibilities analyzed, the “intermediate” susceptibility category was considered nonsusceptible for clinical purposes.

The antibiotic therapy administered was considered appropriate when the isolated pathogen was susceptible to at least one of the antibiotics used empirically in the first 48–72 h.

Mortality was assessed within 7 and 30 days following the bacterial BSI diagnosis; 7-day mortality was considered as associated with the bacterial BSI episode.

### Microbiology laboratory procedures

The blood cultures were processed in the Bactec system (Becton Dickinson) from 2012 to 2021, and BactAlert (BioMérieux) from 2022 to 2023. Bacteria were identified according to routine laboratory procedures, using matrix-assisted laser desorption ionization-time of flight mass spectrometry (MALDI-TOF MS) (Bruker Daltonics). Antimicrobial susceptibility testing (AST) was performed using the disk diffusion method according to CLSI guidelines [25].

### Statistical analysis

Patient demographics and clinical characteristics, as well as microbiological data, were reported as counts and percentages for categorical variables, and as median with interquartile range (IQR) for continuous variables. Categorical values and proportions were assessed using the chi-square test or Fisher’s exact test. For all analyses, two-sided *p*-values < 0.05 were considered statistically significant. All statistical analyses were performed using R Statistical Software (version 4.5.2; R Foundation for Statistical Computing, Vienna, Austria).

## Results

### Demographics and epidemiology

In total, 775 bacterial BSI episodes were identified in 456 patients, with a median age of 5.51 years (Table 1). There were 273 (59.87%), 101 (22.15%) and 47 (10.31%) patients with 1, 2, or 3 bacterial BSI episodes. There were 292 (63.04%) patients with hematologic malignancies (mainly acute lymphoblastic leukemia and acute myeloid leukemia) and 164 (35.96%) with solid tumors (mainly neuroblastoma and primary brain/central nervous system (CNS) tumors).

**Table 1:** Patient characteristics (456 children with 775 bacterial BSI episodes during 2012–2023)

Characteristic	Patients with bacterial-BSI
**Gender**	
Male (*n*, %)	249 (54.61)
Female (*n*, %)	207 (45.39)
**Age groups at BSI episode:**	
< 1 years (*n*, %)	54 (6.97)
0–3 years (*n*, %)	279 (36.00)
4–7 years (*n*, %)	211 (27.23)
8–12 years (*n*, %)	128 (16.52)
13–17 years (*n*, %)	157 (20.26)
Age at BSI episode (years, median [IQR])	5.51 [2.63–10.83]
**Comorbidities (*n*, %)**	54 (11.84)
Central nervous system (*n*, %)	17 (31.48)
Infectious (*n*, %)	11 (20.37)
Respiratory (*n*, %)	6 (11.11)
Hematology (*n*, %)	5 (9.26)
Cardiovascular (*n*, %)	5 (9.26)
Other (*n*, %)	10 (18.52)
**No. of patients with BSI episodes (*n*)**	456
Patients with 1 episode (*n*, %)	273 (59.87)
Patients with 2 episodes (*n*, %)	101 (22.15)
Patients with 3 episodes (*n*, %)	47 (10.31)
Patients with > 3 episodes (*n*, %)	35 (7.67)
**Hematologic malignancies (*n*, %)**	292 (64.03)
Acute lymphoblastic leukemia (*n*, %)	160 (54.79)
Acute myeloid leukemia (*n*, %)	75 (25.68)
Non-Hodgkin’s lymphoma (*n*, %)	20 (6.85)
Hodgkin’s lymphoma (*n*, %)	13 (4.45)
Burkitt lymphoma (*n*, %)	13 (4.45)
Myelodysplastic syndrome (*n*, %)	5 (1.71)
Myelofibrosis (*n*, %)	1 (0.34)
Other (*n*, %)	5 (1.71)
**Solid tumors (*n*, %)**	164 (35.96)
Neuroblastoma (*n*, %)	45 (27.44)
Primary brain/CNS tumors (*n*, %)	35 (21.34)
Rhabdomyosarcoma and nonrhabdomyosarcoma soft tissue sarcomas (*n*, %)	30 (18.29)
Wilm’s tumor (*n*, %)	15 (9.15)
Ewing sarcoma (*n*, %)	13 (7.93)
Retinoblastoma (*n*, %)	10 (6.10)
Osteosarcoma (*n*, %)	3 (1.83)
Hepatoblastoma (*n*, %)	3 (1.83)
Other (n, %)	10 (6.10)
Anti-PCP prophylaxis (TMP/SMX) (*n*, %)	456 (100.0)
Hematopoietic stem cell transplant (*n*, %)	36 (7.89)

Background disease history/comorbidities were reported in 44 (9.65%) of the patients, the majority represented by central nervous system conditions (38.64% of all comorbidities, of them 5 patients with ataxia-telangiectasis, 4 facial nerve paralysis, and 2 epilepsy cases); infectious conditions (25%, 2 zoster, 2 meningitis, and 2 sinusitis cases); respiratory conditions (13.64%, 3 bronchiolitis and 2 pneumonia); and hematological nononcologic conditions (11.36%, 3 thrombocytopenia and 2 Pearson disease). Three (0.6%) patients suffered from Down’s syndrome.

Overall, 297 (38.32%) episodes had a fever ≥ 38.0 °C at admission and 151 (19.48%) had a fever ≥ 39.0 °C (Table 2). Neutropenia ≤ 0.5 × 10^9^/L was recorded in 446 (57.54%) episodes and profound neutropenia (absolute neutrophil count (ANC) ≤ 0.1 × 10^9^/L) in 362 (46.71%) of the episodes. Fever ≥ 38.0 °C and neutropenia ≤ 0.5 × 10^9^/L occurred in 381 (49.16%) of all episodes, with a median patient age of 6.49 (IQR 3.55–12.42). Fever ≥ 38.0 °C without neutropenia ≤ 0.5 × 10^9^/L was recorded in 229 (29.55%) episodes, with a median patient age of 4.87 (IQR 2.43–8.48). Neutropenia ≤ 0.5 × 10^9^/L without fever ≥ 38.0 °C occurred in 72 (9.29%) episodes with a median patient age of 6.40 (IQR 2.48–13.58).

**Table 2:** Laboratory, imaging, and outcome (456 patients with 775 bacterial-BSI episodes)

Characteristic	Patients with bacterial-BSI
Fever at time of culture:	
Temperature ≥ 38.0˚C (*n*, %)	610 (78.71)
Fever ≥ 38.5˚C (*n*, %)	297 (38.32)
Fever ≥ 39.0˚C (*n*, %)	151 (19.48)
Fever (≥ 38.0˚C) without neutropenia (ANC* ≤ 0.5 × 10^9^/L) (n, %)	229 (29.55)
Neutropenia (ANC* ≤ 0.5 × 10^9^/L) (*n*, %)	446 (57.55)
Profound neutropenia (ANC* ≤ 0.1 × 10^9^/L) (*n*, %)	362 (46.71)
Neutropenia (ANC* ≤ 0.5 × 10^9^/L) without fever(≥ 38.0˚C):	72 (9.29)
Fever (≥ 38.0˚C) and neutropenia (ANC* ≤ 0.5 × 10^9^/L) at time of culture (*n*, %):	381 (49.16)
Chest X-rays at time of culture**	
Normal (*n*, %)	225 (61.98)
New alveolar infiltrates (*n*, %)	78 (21.49)
Pleural effusion (*n*, %)	33 (9.09)
New diffuse interstitial infiltrates (*n*, %)	32 (8.82)
Septic shock (*n*, %)	81 (10.45)
PICU admission*** (*n*, %)	230 (29.68)
Mechanical ventilation*** (*n*, %)	55 (7.10)
Death	
7-day all-cause mortality (*n*, %)	29 (6.36)
30-day all-cause mortality (*n*, %)	67 (14.69)

^*^ANC refers to absolute neutrophil count

^**^Information was available in 363 bacterial-BSI episodes

^***^During first 14 days from positive culture

Chest X-rays were performed at admission in 363 episodes; a new alveolar infiltrate and a new diffuse interstitial infiltrate were diagnosed in 78/363 (21.49%) and 32/363 (8.82%) of examinations. A viral co-infection was diagnosed in 42 episodes (cytomegalovirus (CMV), Epstein–Barr virus (EBV), respiratory syncytial virus (RSV), varicella, and rhinovirus in 14, 7, 7, 7, and 6 patients, respectively).

### Blood culture results

A total of 849 pathogens were identified in 775 bacterial BSI episodes (Table 3). There were 584 (68.78%) GN pathogens, 262 (30.86%) GP pathogens, and 3 (0.4%) *Mycobacterium* spp. Monomicrobial bacterial BSI accounted for 706 (91.10%) episodes, while polymicrobial bacterial BSI accounted for 69 episodes (8.90%).

**Table 3:** Microbiological data (849 pathogens recovered in 775 bacterial BSI episodes)

Total pathogens		Part of polymicrobial BSI
**Gram-negative bacteria (*n*, % of all pathogens)**	584 (68.78)*	
*Klebsiella* spp.	173 (20.38)	35 (20.23)**
*Escherichia coli*	163 (19.20)	31 (19.02)
*Pseudomonas aeruginosa*	102 (12.01)	21 (20.59)
*Enterobacter* spp.	53 (6.24)	8 (15.09)
*Acinetobacter baumannii*	14 (1.65)	3 (21.43)
*Citrobacter* spp.	14 (1.65)	7 (50.0)
*Stenotrophomonas maltophilia*	13 (1.53)	5 (38.46)
*Salmonella non-typhi* spp.	13 (1.53)	1 (7.69)
*Serratia* spp.	7 (0.82)	3 (42.86)
*Proteus mirabilis*	6 (0.71)	1 (16.67)
*Moraxella catarrhalis*	5 (0.59)	0 (0.0)
*Bacteroides* spp.	4 (0.47)	0 (0.0)
Other	17 (2.00)	2 (11.76)
**Gram-positive bacteria (*n*, %)**	**262 (30.86)**	
*Streptococcus viridans* spp.	78 (9.19)	4 (5.13)
*Enterococcus* spp.	54 (6.36)	14 (25.93)
*Staphylococcus aureus*	52 (6.12)	5 (9.62)
Coagulase-negative Staphylococcus	44 (5.18)	0 (0.0)
*Streptococcus pneumoniae*	17 (2.00)	1 (5.88)
*Abiotrophia defectiva*	5 (0.59)	1 (20.00)
*Clostridium* spp.	4 (0.47)	0 (0.0)
*Streptococcus pyogenes*	3 (0.35)	0 (0.0)
*Bacillus cereus*	3 (0.36)	0 (0.0)
*Streptococcus dysgalactiae*	2 (0.24)	1 (50.00)
**Mycobacterium spp.**	**3 (0.4)**	**0 (0.0)**

^*^Percentage of total number of isolated pathogens

^**^Percentage of total isolates of each pathogen

The most frequent GN pathogens were *Klebsiella* spp. (20.38% of all pathogens), followed by *Escherichia coli* (19.20%) and *Pseudomonas aeruginosa* (12.10%). The *Enterobacteriaceae* group represented 50.53% of all pathogens and 73.46% of all GN pathogens. Among GN pathogens, *Klebsiella* spp., *E. coli*, and *P. aeruginosa* represented 29.62%, 27.91%, and 17.47% of all isolates. The most common GP pathogens were *Streptococcus viridans* spp. (9.19% of all isolates), *Enterococcus* spp. (6.36%), and *Staphylococcus aureus* (6.12%). Among GP pathogens, *S. viridans* spp., *Enterococcus* spp., and *S. aureus* represented 29.77%, 20.61%, and 19.85 of all isolates, respectively.

**Fig. 1:**
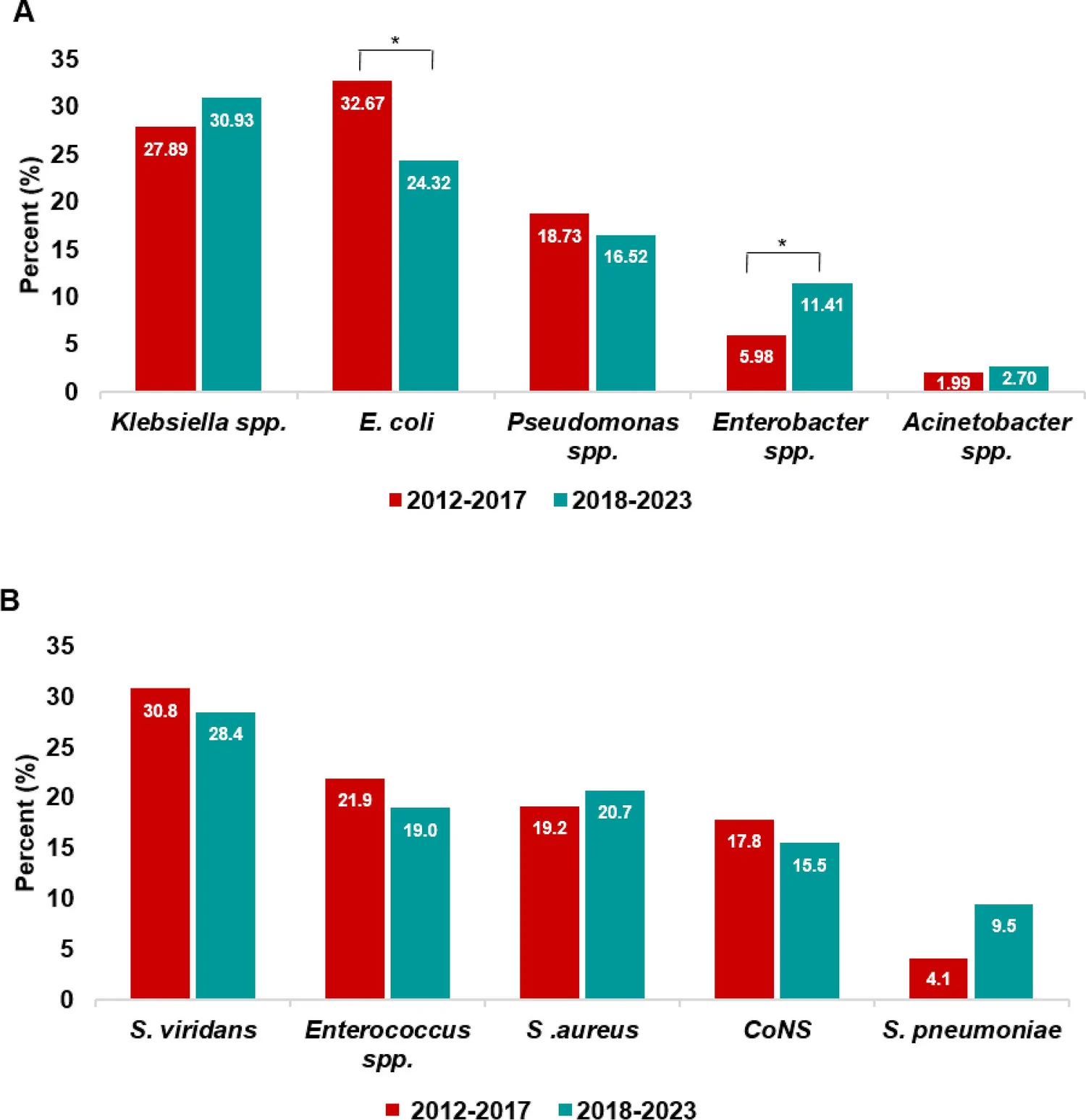
Distribution of **A** gram-negative and **B** gram-positive pathogens during the two study periods (2012–2017 and 2018–2023).^*^p ≤ 0.05 and.^**^p ≤ 0.01

Overall, 397 pathogens were isolated during the first 6 years of the study (2012–2017) and 449 (a 13.1% increase) during the last 6 years of the study (2018–2023). An increase of 17.30% in the percentages of GN pathogens was observed between the two study periods (251/397, 63.22% vs. 333/449, 74.16%, *P* = 0.001), accompanied by a decrease of 29.74% in the percentages of GP pathogens (146/397, 36.78% vs. 116/449, 25.84%, *P* = 0.001) (Fig. 1). An increase in *Enterobacter* spp. rates (15/251, 5.98%, vs. 38/333, 11.41%, *P* = 0.024) among all GN pathogens was observed in the second study period. A decrease in the percentage of *E. coli* (82/251, 32.67%, vs. 81/333, 24.32%, *P* = 0.026) among all GN pathogens was observed in the second study period. No differences between the two study periods were recorded in the percentages of *Klebsiella* spp., *P. aeruginosa*, and *A. baumannii*. Regarding GP pathogens, no statistically significant differences were observed between the two study periods (Fig. 1).

### Antibiotic nonsusceptibility

The nonsusceptibility to antibiotics was analyzed for 706/849 (83.16%) pathogens, including the most common five GN (*Klebsiella* spp., *E. coli*, *P. aeruginosa*., *Enterobacter* spp., and *A. baumannii*; total 505 pathogens) and four GP (*S. viridans*, *Enterococcus* spp., *S. aureus*, and *S. pneumoniae*; total 201 pathogens) pathogens.

Overall nonsusceptibility rates of *Klebsiella* spp. and *E. coli* were 80.13% and 90.07% for piperacillin, 16.76% and 9.82% for amikacin, 58.23% and 50.92% for ciprofloxacin, 69.19% and 50.31% for ceftriaxone, 34.68% and 23.92% for piperacillin/tazobactam, and 8.72% and 1.85% for meropenem. ESBLs were identified in most evaluable *Klebsiella* spp. and of *E. coli* isolates (98.10% and 100.00%, respectively). Nonsusceptibility rates of *P. aeruginosa*. to piperacillin, amikacin, ciprofloxacin, piperacillin/tazobactam and meropenem were 8.91%, 2.97%, 5.00, 8.91%, and 5.00%, respectively. The nonsusceptibility rates of *Enterobacter* spp. to piperacillin/tazobactam and meropenem were 19.23% and 5.66%, respectively, while the respective rates of nonsusceptibility of *A. baumanii* were 57.14% and 57.14%, respectively.

Among GP isolates, 4.20% (11/262, 21.15% of all *S*. *aureus* isolates) were MRSA; 67.95%, 29.49%, and 59.21% *S. viridans* isolates were nonsusceptible to penicillin, ceftriaxone, and erythromycin. Ten *S. pneumoniae* isolates were resistant to penicillin and all seventeen isolates were sensitive to ceftriaxone. Of 31 *E. faecium* isolates, 4 (12.90%) were vancomycin-resistant.

### Antibiotic nonsusceptibility dynamics (2012–2017 vs. 2018–2023)

**Fig. 2:**
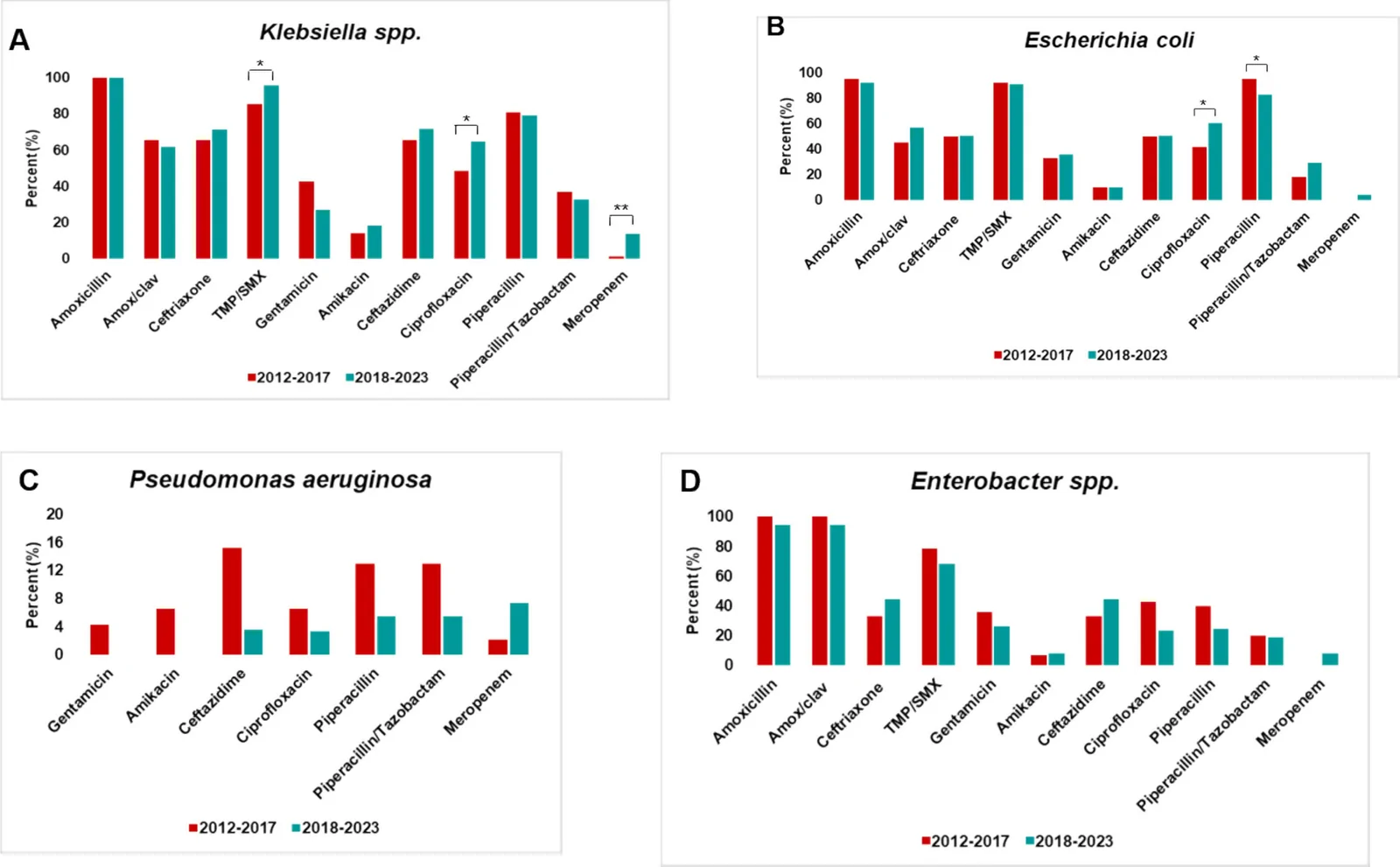
Nonsusceptibility of Gram-negative pathogens during the two study periods (2012–2017 and 2018–2023): **A** *Klebsiella* spp., **B** *Escherichia coli*, **C** *Pseudomonas aeruginosa*, and **D** Enterobacter spp. ^*^p ≤ 0.05 and.^**^p ≤ 0

Between the two study periods, an increase was observed in the nonsusceptibility rates of *K. pneumoniae* to ciprofloxacin (48.57 to 65.00%, *P* = 0.03) and meropenem (1.43 to 13.73%, *P* = 0.001), and in the nonsusceptibility rates of *E. coli* to ciprofloxacin (41.46 to 60.49%, *P* = 0.02); the *E. coli* isolates showed an increasing trend for nonsusceptibility to piperacillin/tazobactam (18.29 to 29.63%, *P* = 0.09) and meropenem (0.00 to 3.70%, *P* = 0.08) (Fig. 2). Among GP pathogens, no changes in MRSA rates were observed during the two periods studied while a decrease was reported in the nonsusceptibility of *S. viridans* to penicillin (77.78% vs. 54.55%, *P* = 0.05) and ceftriaxone (42.22% vs. 12.12%, *P* = 0.04).

### Empiric antibiotic treatment (EAT)

EAT appropriateness was analysed in 689/849 (81.15%) evaluable pathogens, including the most common five GN (*Klebsiella* spp., *E. coli*, *P. aeruginosa*., *Enterobacter* spp., and *A. baumannii*; total 500 pathogens) and four GP (*S. viridans*, *Enterococcus* spp., *S. aureus,* and *S. pneumoniae*; 189 pathogens) pathogens. No EAT was administered in 15 episodes.

The most frequent choices of EAT were piperacillin/tazobactam plus amikacin (199/689 isolates, 28.88%), piperacillin/tazobactam (115, 16.69%), piperacillin plus amikacin (90, 13.06%), and meropenem (77, 11.18.2%). Vancomycin was used for 60 isolates (8.71%, as single therapy for 3 isolates and as part of double antibiotic therapy for 41).

### EAT appropriateness

The EAT was appropriate for 531/689 (77.07%) evaluable pathogens, of them for 408/500 (81.6%) GN and 124/189 (65.61%) GP pathogens. The appropriateness of therapy was 75.59%, 82.1%, 93.0%, 88.68%, and 53.85% for *Klebsiella* spp., *E. coli*, *P. aeruginosa*, *Enterobacter* spp., and *A. baumannii*, respectively.

The most frequently used EAT (piperacillin/tazobactam plus amikacin) was found appropriate in 180/199 (90.45%) treatments. The appropriateness of EAT with piperacillin/tazobactam alone was significantly lower (72/115, 62.61%), *P* < 0.0001.

### Patient outcomes

PICU admissions were required in 230 (29.68%, 252 pathogens) episodes, and mechanical ventilation was reported in 55 (7.10%) episodes. The four most common recovered pathogens in patients admitted to PICU were: *E. coli* (61/252, 24.21%), *K. pneumoniae* (59/252, 23.41%), *P. aeruginosa* (28/252, 11.11%), and *Enterococcus* spp. (18/252, 7.14%, 9 *E. faecium*).

Septic shock occurred in 81 (10.45%) BSI episodes (monobacterial in 73 episodes and polybacterial in 8 episodes). Five of these polymicrobial BSI episodes were caused by *Klebsiella* spp. plus *E. coli*. From a total of 88 pathogens (77 GN, 11 GP) isolated in patients with septic shock, the most common were *K. pneumoniae* and *E. coli* (28/88, 31.82% and 27/88, 30.68%) isolates, followed by *P. aeruginosa* (8, 9.09%) and *Enterobacter* spp. (7, 7.95%). Overall, 10.81% of the GN isolates responsible for septic shock (8/74 checked) were nonsusceptible to meropenem. The respective nonsusceptibility rates to piperacillin/tazobactam, amikacin, piperacillin/tazobactam plus amikacin, and meropenem plus amikacin were 24.32% (18/74), 17.57% (13/74), 13.51% (10/74), and 8.1% (6/74), respectively.

Death occurring during the first 7 days was recorded in 29/456 (6.36%, 32 pathogens) of patients. The three most common recovered pathogens in fatalities within 7 days were *K. pneumoniae* (10/32, 31.25%), *Enterococcus* spp. (4/32, 12.5%), and *P. aeruginosa* (4/32, 12.5%). The 30-day mortality rate was 14.69% (67/456).

## Discussion

Recent information on the microbiological characteristics, antibiotic resistance patterns, therapeutic protocols, and outcomes in pediatric patients hospitalized in hematology-oncology units in Israel is limited. In Jerusalem, Vinker-Shuster et al. [26] characterized 85 BSI episodes caused by gram-negative–resistant pathogens in 270 hemato-oncologic and post-HSCT children admitted during 2010–2014. Overall, 10% of the pathogens were carbapenem-resistant. Carbapenem-resistant vs. carbapenem-sensitive bacteremia was associated with longer duration of bacteremia, higher risk for PICU admission, and higher mortality rates. Arad-Cohen et al. [27] reported on GN bacteremias among 62 children with de novo acute myelocytic leukemia; 77.4% had 1–8 bacteremia episodes. Of 238 chemotherapy courses, 98 (41.2%) developed bacteremia episodes: 66 (67.3%) isolates were GN rods (mostly *E. coli* and *Klebsiella* spp.), mortality was high (9.7%), and most isolates were sensitive to vancomycin plus meropenem (94.75%).

Our retrospective study enrolled a considerable number of children with cancer and bacterial BSIs over a 12-year period. We found that 21.29% episodes were afebrile at admission and profound neutropenia was reported in 46.71% of the episodes. The proportion of neutropenic children in this study was comparable with other studies [13, 14, 20–22, 26, 28]. In addition, a new alveolar infiltrate and a new diffuse interstitial infiltrate were diagnosed in 21.49% and 8.82% of patients in whom X-rays examinations were performed at admission, emphasizing the involvement of respiratory infections in the etiology of BSIs in hemato-oncologic patients. GN pathogens represented 68.78% of all pathogens, with a significant increase to 77.26% during the last 6 years of the study. These findings are in accordance with recent reports emphasizing the return of GNs predominance in most BSIs in children with cancer [3, 4, 14, 18, 19]. We showed that most common GN pathogens were represented by *Klebsiella* spp., *E. coli*, and *P. aeruginosa,* and the *Enterobacteriaceae* spp. group represented 73.46% of all GN pathogens. The most common GP pathogens were represented by *S. viridans*, *Enterococcus* spp., and *S. aureus.* We found an increase in *Enterobacter* spp. rates isolated in the second study period while *Klebsiella* spp.* P. aeruginosa*,* A. baumannii*, and *S. aureus* recovery rates were stable during the study period.

Among GN pathogens, resistance to carbapenems in infections caused by *K. pneumoniae* and *Acinetobacter* spp. has recently been reported as one of the major emerging causes of severe and fatal infections in hemato-oncology patients [29–35]. We reported high (> 55%) nonsusceptibility rates of *Klebsiella* spp. and *E. coli* to piperacillin, ceftriaxone, and ciprofloxacin and moderately high nonsusceptibility rates (34.68% and 23.92%) to piperacillin/tazobactam. ESBLs were identified in most *Klebsiella* spp. and of *E. coli* isolates. We also showed relatively high nonsusceptibility rates (57.14% and 57.14%, respectively) to piperacillin/tazobactam and meropenem among *A. baumannii* isolates. Among the GP pathogens, 21.15% of *S. aureus* isolates were MRSA and the nonsusceptibility of *S*. *viridans* of penicillin, ceftriaxone, and erythromycin was high. These findings are of concern and are similar to data previously reported in the medical literature, although major differences were observed between different countries [13–17, 27–29].

The most frequent EAT used were piperacillin/tazobactam plus amikacin, piperacillin/tazobactam alone, piperacillin plus amikacin, and meropenem. Recent guidelines for the management of fever and neutropenia in pediatric cancer patients recommend monotherapy with an antipseudomonal penicillin, such as piperacillin-tazobactam, or a fourth-generation cephalosporin or a carbapenem, as EAT. No difference in treatment outcome has been described between the suggested antibiotics [3, 4, 19, 20, 28]. However, we observed relatively high nonsusceptibility rates to piperacillin/tazobactam among the main GN pathogens and therefore cannot recommend, in our setting, use of this drug alone as the first-line EAT. We consider that our antibiotic nonsusceptibility findings are worrisome, particularly in respect to ceftazidime and piperacillin/tazobactam nonsusceptibility rates vs. *Klebsiella* spp. and *E*. *coli*, which may result in an increased risk of treatment failure [13, 14, 17, 19, 20, 28, 29, 31]. During the last years of the study, an EAT was used with a β-lactam plus β-lactamase inhibitor combination (piperacillin/tazobactam) plus amikacin, for clinically stable patients. The first-line EAT combination (piperacillin/tazobactam plus amikacin) was found to cover the majority (90.45%) of pathogens causing bacterial BSIs at our medical center. Carbapenems, such as meropenem, were usually reserved for treatment of more resistant pathogens, clinically unstable patients, and as EAT in sites with a high prevalence of resistant GN [3, 4, 20, 28]. For clinically unstable patients, even when at low risk of infection with resistant pathogens, patients with septic shock, those with previous bacterial BSIs or previously colonized with multidrug-resistant pathogens or as a second line of therapy after failure of first-line therapies, we recommended the use of an empiric treatment with piperacillin/tazobactam, meropenem, or meropenem plus amikacin, with the possible addition of vancomycin.

This study has some limitations. First, we report a single-center experience, which limits the generalizability of our findings. To balance this limitation, we assessed a large cohort of 838 BSIs through 12 years in children with a wide spectrum of malignancies. Second, the retrospective study design might have missed some clinical, laboratory, and treatment characteristics and may have increased the risk of bias in some cases.

In conclusion, we reported an overall dominance of GN-BSI isolates during the study period with an increase in GN-BSIs rates during the last 6 years of the study. We reported increased antimicrobial resistance rates of *Klebsiella* spp. and *E. coli* against piperacillin/tazobactam and of *A. baumannii* against meropenem. In our setting, use of an EAT protocol including piperacillin/tazobactam plus amikacin was found to cover the majority of pathogens isolated in the study patients.

## Data Availability

The datasets generated during and/or analyzed during the current study are available from the corresponding author on reasonable request.

## Abbreviations

ANC: Absolute neutrophil count
ALL: Acute lymphoblastic leukemia
AML: Acute myeloid leukemia
BSIs: Bloodstream infections
CNS: Central nervous system
CMV: Cytomegalovirus
EAT: Empiric antibiotic therapy
EBV: Epstein–Barr virus
ESBL: Extended-spectrum β-lactamase-producing
GN: Gram-negative
GP: Gram-positive GP
MDR: Multidrug-resistant
HSCT: Hematopoietic stem cell transplant
IQR: Interquartile range
RSV: Respiratory syncytial virus

## References

[1] Hakim H, Flynn PM, Knapp KM, Srivastava DK, Gaur AH (2009) Etiology and clinical course of febrile neutropenia in children with cancer. J Pediatr Hematol Oncol 31(9):623–629. 10.1097/MPH.0b013e3181b1edc6 PMC2743072 19644403

[2] Radhakrishnan V, Vijaykumar V, Ganesan P, Rajendranath R, Trivadi G, Tenali S (2014) Bloodstream infections in pediatric patients at Cancer Institute, Chennai. Indian J Cancer 51(4):418–419. 10.4103/0019-509X.175360 26842144

[3] Lehrnbecher T, Robinson P, Fisher B, Alexander S, Ammann RA, Beauchemin M et al (2017) Guideline for the management of fever and neutropenia in children with cancer and hematopoietic stem-cell transplantation recipients: 2017 update. J Clin Oncol 35(18):2082–2094. 10.1200/JCO.2016.71.7017 28459614

[4] Lehrnbecher T, Averbuch D, Castagnola E, Cesaro S, Ammann RA, Garcia-Vidal C et al (2021) 8th European Conference on Infections in Leukaemia: 2020 guidelines for the use of antibiotics in paediatric patients with cancer or post-haematopoietic cell transplantation. Lancet Oncol 22(6):e270–e280. 10.1016/S1470-2045(20)30725-7 33811814

[5] Ammann RA, Laws HJ, Schrey D, Ehlert K, Moser O, Dilloo D et al (2015) Bloodstream infection in paediatric cancer centres-leukaemia and relapsed malignancies are independent risk factors. Eur J Pediatr 174(5):675–686. 10.1007/s00431-015-2525-5 25804192

[6] Lehrnbecher T, Robinson PD, Ammann RA, Fisher B, Patel P, Phillips R et al (2023) Guideline for the management of fever and neutropenia in pediatric patients with cancer and hematopoietic cell transplantation recipients: 2023 update. J Clin Oncol 41(9):1774–1785. 10.1200/JCO.22.02224 PMC10022858 36689694

[7] Thurman CB, Abbott M, Liu J, Larson E (2017) Risk for health care-associated bloodstream infections in pediatric oncology patients with various malignancies. J Pediatr Oncol Nurs 34(3):196–202. 10.1177/1043454216680596 PMC6711581 28038498

[8] Haeusler GM, Phillips R, Slavin MA, Babl FE, De Abreu LR, Mechinaud F et al (2020) Re-evaluating and recalibrating predictors of bacterial infection in children with cancer and febrile neutropenia. EClinicalMedicine 23:100394. 10.1016/j.eclinm.2020.100394 PMC7329706 32637894

[9] Castagnola E, Caviglia I, Pistorio A, Fioredda F, Micalizzi C, Viscoli C et al (2005) Bloodstream infections and invasive mycoses in children undergoing acute leukaemia treatment: a 13-year experience at a single Italian institution. Eur J Cancer 41(10):1439–1445. 10.1016/j.ejca.2005.03.007 15963894

[10] Sung L, Gamis A, Alonzo TA, Buxton A, Britton K, Deswarte-Wallace J et al (2009) Infections and association with different intensity of chemotherapy in children with acute myeloid leukemia. Cancer 115(5):1100–1108. 10.1002/cncr.24107 PMC2677372 19156894

[11] Garrido MM, Garrido RQ, Cunha TN, Ehrlich S, Martins IS (2019) Comparison of epidemiological, clinical and microbiological characteristics of bloodstream infection in children with solid tumors and haematological malignancies. Epidemiol Infect 8(147):e298. 10.1017/S0950268819001845 PMC6873156 31699182

[12] Miedema KGE, Winter RHLJ, Ammann RA, Droz S, Spanjaard L, de Bont ESJM et al (2013) Bacteria causing bacteremia in pediatric cancer patients presenting with febrile neutropenia–species distribution and susceptibility patterns. Supp Care Cancer 21(9):2417–2426. 10.1007/s00520-013-1797-4 23579946

[13] Castagnola E, Caviglia I, Pescetto L, Bagnasco F, Haupt R, Bandettini R (2015) Antibiotic susceptibility of gram-negatives isolated from bacteremia in children with cancer. Implications for empirical therapy of febrile neutropenia. Future Microbiol. 10(3):357–364. 10.2217/fmb.14.144 25812459

[14] Lee JH, Kim SK, Kim SK, Han SB, Lee JW, Lee DG et al (2016) Increase in antibiotic-resistant gram-negative bacterial infections in febrile neutropenic children. Infect Chemother 48(3):181–189. 10.3947/ic.2016.48.3.181 PMC5047999 27659431

[15] Knight T, Glaser DW, Ching N, Melish M (2019) Antibiotic susceptibility of bloodstream isolates in a pediatric oncology population: the case for ongoing unit-specific surveillance. J Pediatr Hematol Oncol 41(5):e271–e276. 10.1097/MPH.0000000000001498 31033794

[16] Caselli D, Cesaro S, Ziino O, Zanazzo G, Manicone R, Livadiotti S et al (2010) Multidrug resistant Pseudomonas aeruginosa infection in children undergoing chemotherapy and hematopoietic stem cell transplantation. Haematologica 95(9):1612–1615. 10.3324/haematol.2009.020867 PMC2930967 20305140

[17] Haeusler GM, Mechinaud F, Daley AJ, Starr M, Shann F, Connell TG et al (2013) Antibiotic-resistant gram-negative bacteremia in pediatric oncology patients–risk factors and outcomes. Pediatr Infect Dis J 32(7):723–726. 10.1097/INF.0b013e31828aebc8 23838774

[18] Islas-Muñoz B, Volkow-Fernández P, Ibanes-Gutiérrez C, Villamar-Ramírez A, Vilar-Compte D, Cornejo-Juárez P (2018) Bloodstream infections in cancer patients. Risk factors associated with mortality. Int J Infect Dis 71:59–64. 10.1016/j.ijid.2018.03.022 29649549

[19] Mikulska M, Viscoli C, Orasch C, Livermore DM, Averbuch D, Cordonnier C et al (2014) Aetiology and resistance in bacteraemias among adult and paediatric haematology and cancer patients. J Infect 68(4):321–331. 10.1016/j.jinf.2013.12.006 24370562

[20] Trecarichi EM, Giuliano G, Cattaneo C, Ballanti S, Criscuolo M, Candoni A et al (2023) Bloodstream infections due to gram-negative bacteria in patients with hematologic malignancies: updated epidemiology and risk factors for multidrug-resistant strains in an Italian perspective survey. Int J Antimicrob Agents 61(6):106806. 10.1016/j.ijantimicag.2023.106806 37030470

[21] Castagnola E, Bagnasco F, Mesini A, Agyeman PKA, Ammann RA, Carlesse F et al (2021) Antibiotic resistant bloodstream infections in pediatric patients receiving chemotherapy or hematopoietic stem cell transplant: factors associated with development of resistance, intensive care admission and mortality. Antibiotics (Basel) 10(3). 10.3390/antibiotics10030266 PMC8000765 33807654

[22] O’Connor D, Bate J, Wade R, Clack R, Dhir S, Hough R et al (2014) Infection-related mortality in children with acute lymphoblastic leukemia: an analysis of infectious deaths on UKALL2003. Blood 124(7):1056–1061. 10.1182/blood-2014-03-560847 24904116

[23] Willis DN, McGlynn MC, Reich PJ, Hayashi RJ (2022) Mortality in pediatric oncology and stem cell transplant patients with bloodstream infections. Front Oncol 12:1063253. 10.3389/fonc.2022.1063253 PMC9874914 36713545

[24] Hall KK, Lyman JA (2006) Updated review of blood culture contamination. Clin Microbiol Rev 19(4):788–802. 10.1128/CMR.00062-05 PMC1592696 17041144

[25] CLSI (2023) Performance standards for antimicrobial susceptibility testing, M100, 33rd ed. Clinical and Laboratory Standards Institute, Wayne, PA

[26] Vinker-Shuster M, Stepensky P, Temper V, Shayovitz V, Masarwa R, Averbuch D (2019) Gram-negative bacteremia in children with hematologic malignancies and following hematopoietic stem cell transplantation: epidemiology, resistance, and outcome. J Pediatr Hematol Oncol 41(8):e493–e498. 10.1097/MPH.0000000000001556 31318820

[27] Arad-Cohen N, Messinger Y, Barzilai-Birenboim S, Ben-Harosh M, Golan-Malki M, Rosenfeld-Keidar H et al (2024) National study reveals gram negative bacteremia on contemporary pediatric AML protocol. Leuk Lymphoma 65(13):1991–1999. 10.1080/10428194.2024.2385496 39078922

[28] Maarbjerg SF, Kiefer LV, Albertsen BK, Schrøder H, Wang M (2022Jan 1) Bloodstream infections in children with cancer: pathogen distribution and antimicrobial susceptibility patterns over a 10-year period. J Pediatr Hematol Oncol 44(1):e160–e167. 10.1097/MPH.0000000000002258. (**PubMed PMID: 34310474**) 34310474

[29] Trecarichi EM, Pagano L, Martino B, Candoni A, Di Blasi R, Nadali G et al (2016) Bloodstream infections caused by Klebsiella pneumoniae in onco-hematological patients: clinical impact of carbapenem resistance in a multicentre prospective survey. Am J Hematol 91(11):1076–1081. 10.1002/ajh.24489 27428072

[30] Averbuch D, Tridello G, Hoek J, Mikulska M, Akan H, Yanez San Segundo L et al (2017) Antimicrobial resistance in Gram-negative rods causing bacteremia in hematopoietic stem cell transplant recipients: intercontinental prospective study of the infectious diseases working party of the European bone marrow transplantation group. Clin Infect Dis 65(11):1819–1828. 10.1093/cid/cix646 29020364

[31] Tofas P, Skiada A, Angelopoulou M, Sipsas N, Pavlopoulou I, Tsaousi S et al (2016) Carbapenemase-producing Klebsiella pneumoniae bloodstream infections in neutropenic patients with haematological malignancies or aplastic anaemia: analysis of 50 cases. Int J Antimicrob Agents 47(4):335–339. 10.1016/j.ijantimicag.2016.01.011 27005460

[32] Jaiswal SR, Gupta S, Kumar RS, Sherawat A, Rajoreya A, Dash SK et al (2018) Gut colonization with carbapenem-resistant Enterobacteriaceae adversely impacts the outcome in patients with hematological malignancies: results of a prospective surveillance study. Mediterr J Hematol Infect Dis 10(1):e2018025. 10.4084/MJHID.2018.025 PMC5937952 29755703

[33] Lalaoui R, Javelle E, Bakour S, Ubeda C, Rolain JM (2020) Infections due to carbapenem-resistant bacteria in patients with hematologic malignancies. Front Microbiol 11:1422. 10.3389/fmicb.2020.01422 PMC7379235 32765433

[34] Biswas S, Bhat V, Kelkar R (2020) Carbapenem-resistant enterobacteriaceae: a serious concern in cancer patients. Access Microbiol 2(1). 10.1099/acmi.mim2019.po0008

[35] Logan LK, Gandra S, Trett A, Weinstein RA, Laxminarayan R (2019) Acinetobacter baumannii resistance trends in children in the United States, 1999–2012. J Pediatric Infect Dis Soc 8(2):136–142. 10.1093/jpids/piy018 PMC6510944 29579216

