# Correction to: Bacterial bloodstream infections in pediatric oncology patients in a tertiary medical center: pathogen distribution, antimicrobial nonsusceptibility, and outcomes

**DOI:** 10.1007/s00431-026-07499-w

**Published:** 2026-10-15

**Authors:** Menucha Jurkowicz, Eugene Leibovitz, Hila Rosental, Nathan Keller, Gilad Sherman, Efrat Dresner, Guy Katzenellenbogen, Elina Gelman, Michal Paret, Reut Myaskovetzky-Yitshack, Hana Golan, Assaf Barg, Amos Toren, Michal Stein

**Affiliations:** 1 Pediatric Infectious Disease Unit, The Edmond and Lily Safra Children’s Hospital, Sheba Medical Center, Ramat Gan, Israel; 2 Department of Epidemiology and Preventive Medicine, School of Public Health, Gray Faculty of Medicine, Tel Aviv University, Tel Aviv, Israel; 3 Faculty of Health Sciences, Ben-Gurion University, Beer Sheva, Israel; 4 Gray Faculty of Medical and Health Sciences, Tel Aviv University, Tel Aviv, Israel; 5 The Department of Health Management, Ariel University, Ariel, Israel; 6 Division of Infectious Diseases, Department of Pediatrics, Boston Children’s Hospital, Harvard Medical School, Boston, MA USA; 7 Clinical Pharmacy Unit, Sheba Medical Center, Ramat Gan, Israel; 8 Division of Pediatric Hematology, Oncology, and Bone Marrow Transplant, Edmond and Lily Safra Children’s Hospital, Sheba Medical Center, Ramat Gan, Israel

**Correction to: European Journal of Pediatrics (2026) 185:661**

**https://doi.org/10.1007/s00431-026-07305-7**

In the original publication of this article, errors were identified in the text definitions of neutropenia and profound neutropenia, as well as formatting inconsistencies in Table 2.

**Section: Definitions and Results**

- **Published form:** absolute neutrophil count < 500 WBC × 10^9^/L
- **Corrected form:** ≤ 0.5 × 10^9^/L
- **Published form:** absolute neutrophil count < 100 WBC × 10^9^/L
- **Corrected form:** ≤ 0.1 × 10^9^/L

**Table 2**

- **Published form:** 109/L (multiple instances missing superscript 9)
- **Corrected form:** 10^9^/L

The original article has been corrected.

